# Hemodynamic Response Pattern of Spatial Cueing is Different for Social and Symbolic Cues

**DOI:** 10.3389/fnhum.2014.00912

**Published:** 2014-11-10

**Authors:** Denise Elfriede Liesa Lockhofen, Harald Gruppe, Christoph Ruprecht, Bernd Gallhofer, Gebhard Sammer

**Affiliations:** ^1^Cognitive Neuroscience at the Centre for Psychiatry, Justus-Liebig-University Giessen, Giessen, Germany; ^2^Department of Psychology, Justus-Liebig-University Giessen, Giessen, Germany; ^3^Bender Institute of Neuroimaging, Justus-Liebig-University Giessen, Giessen, Germany

**Keywords:** gaze cueing, arrow cueing, functional magnetic resonance imaging, functional connectivity, visual attention, spatial orienting

## Abstract

Directional social gaze and symbolic arrow cues both serve as spatial cues, causing seemingly reflexive shifts of an observer’s attention. However, the underlying neural substrates remain a point at issue. The present study specifically addressed the differences in the activation patterns associated with non-predictive gaze and arrow cues, placing special emphasis on brain regions known to be involved in the processing of social information [superior temporal sulcus (STS), fusiform gyrus (FFG)]. Additionally, the functional connectivity of these brain regions with other areas involved in gaze processing and spatial attention was investigated. Results indicate that gaze and arrow cues recruit several brain regions differently, with gaze cues increasing activation in occipito-temporal regions and arrow cues increasing activation in occipito-parietal regions. Specifically, gaze cues in contrast to arrow cues enhanced activation in the FFG and the STS. Functional connectivity analysis revealed that during gaze cueing the STS was more strongly connected to the intraparietal sulcus (IPS) and the frontal eye fields, whereas the FFG was more strongly connected to the IPS and the amygdala.

## Introduction

It has been widely acknowledged that eye gaze serves as a cue for spatial attention, inducing seemingly reflexive shifts of an observers’ attentional focus (Friesen and Kingstone, 1998; Driver et al., 1999; Friesen et al., 2004, 2005). These shifts of attention occur even when the gaze cue is non-predictive of the target and when the time delay between cue and target is very short (105 ms; Friesen and Kingstone, 1998).

It was assumed that this is due to the special status of eye gaze as a stimulus of social and biological relevance (Friesen and Kingstone, 1998). In contrast to earlier research (Jonides, 1981), recent work has also shown reflexive attention shifts for central symbolic stimuli such as arrows (Ristic et al., 2002; Tipples, 2002), challenging the special role of eye gaze. With few exceptions [e.g., Friesen et al. (2004)], eyes and arrows appear to have similar effects on attention orienting on a behavioral level (Ristic et al., 2002), showing the “same time course, same magnitude, and same gender difference” (Bayliss et al., 2005, 646 p). Nevertheless, only gaze cues have been shown to modulate the affective response to cued objects (although both cue types were able to induce similar cueing effects; Bayliss et al., 2006) and to influence memory performance (Dodd et al., 2012). Since the behavioral results of social gaze and symbolic arrow cueing appear mostly identical, considerable effort has been made to define the underlying neural systems and to determine if eye gaze cues are processed by gaze-specific mechanisms.

Some of these studies found that gaze and arrow cues activated mostly overlapping brain regions. Tipper et al. (2008) utilized an ambiguous stimulus that could be perceived as an eye or as an arrowhead. In their study, participants had to switch the perception of the cue according to the instruction. The results demonstrated similar BOLD-responses for both cue types, though the gaze cue recruited ventral frontal and lateral occipital regions more strongly than the arrow cue. Another study found that the same brain regions were recruited in the processing of eyes, hands, and arrows (Sato et al., 2009). Prior to the main experiment of the study, it was shown that all three stimulus types induced reflexive shifts of attention toward a target stimulus, and thus, serve as a spatial cue. In the following experiment, the participants had to passively view directional and non-directional eyes, hands, and arrows while engaging in a dummy task. A cognitive conjunction analysis showed that on the right hemisphere the superior temporal sulcus (STS), the inferior parietal lobule, the inferior frontal gyrus, and the occipital cortices were commonly activated across stimulus types. Differences in neural activity between the three stimulus types were detected in response to directional versus non-directional eyes (amygdala) and arrows (right posterior temporal cortices and the left superior parietal lobule), but were less clear, probably because of the task-type, which required the participants to passively watch the stimuli (Sato et al., 2009).

In contrast to these studies, others have found that gaze and arrow cues are processed by different brain regions. Hietanen et al. (2006) compared attention orienting elicited by centrally presented schematic eyes and arrows. They contrasted directional (averted gaze, laterally pointing arrows) with non-directional (direct gaze, segment of a line) cues. The behavioral results showed a similar cueing effect for gaze and arrow cues, whereas the imaging results revealed overlapping networks in posterior occipito-temporal regions that were activated to a wider extent by arrow cues, and also several areas that uniquely reacted to arrows (Hietanen et al., 2006). Another study (Kingstone et al., 2004) showed that the STS responded specifically if participants saw an ambiguous stimulus as a pair of eyes instead of a car, even though the behavioral responses to both percepts were similar.

The STS has been linked to the processing of several types of biological motion, including eye movements (Allison et al., 2000), as well as theory of mind, i.e., inferring the intentions of others by social cues (Gallagher and Frith, 2003). STS seems to react specifically to eye motion that provides socially meaningful information (Hooker et al., 2003; Materna et al., 2008). Studies suggest that it might be sensitive to the context in which gaze shifts occur (Pelphrey et al., 2003), as well as to the intentions conveyed by directional eye movements (Mosconi et al., 2005). Most interestingly, it has been demonstrated that the STS reacts stronger to gaze shifts than to directional arrow cues (Hooker et al., 2003). The STS has also been found to be involved in theory of mind, speech processing, audiovisual integration, and face processing (Hein and Knight, 2008). It is a matter of some debate whether the multifunctionality of the STS region is based on strict topographical subdivision within this area or rather on dynamic formation of distributed functional networks, dependent on coactivated remote regions subserving a certain behavioral task (cf. Hein and Knight, 2008; Frühholz and Grandjean, 2013). The latter idea is supported by considerable topographical overlap with respect to distinct functions within the STS. It can be addressed by investigating the functional or effective connectivity of the STS with other brain regions.

In addition to the STS, there are several other brain regions that have been linked to the processing of gaze cues. An area that has been associated primarily to the processing of faces (Haxby et al., 2002), especially to the processing of invariant facial features such as face identity (Hoffman and Haxby, 2000), is the fusiform gyrus (FFG). However, there is evidence that this region is also involved in gaze processing (George et al., 2001; Pelphrey et al., 2003; Mosconi et al., 2005; Nummenmaa and Calder, 2009; Nummenmaa et al., 2010). In addition, it has been shown that the connectivity of STS and FFG changes in response to averted gaze or gaze shifts (George et al., 2001; Nummenmaa et al., 2010). The amygdala is supposed to be part of a wider network for face perception, together with the STS and the FFG (Haxby et al., 2000). It has also been linked to gaze processing (Wicker et al., 1998; Kawashima et al., 1999; Nummenmaa and Calder, 2009) and might be important for gaze monitoring in situations in which direct gaze is expected (Hooker et al., 2003). Amygdala lesion leads to impaired attention orienting in response to gaze, but not arrow cues (Akiyama et al., 2007). The frontal eye fields (FEFs) and the intraparietal sulcus (IPS) have established roles as parts of the dorsal attention system (Corbetta and Shulman, 2002) and might be involved in voluntary attentional control (Kincade et al., 2005). Like the amygdala, the IPS, and possibly also the FEF, are part of an extended network for face perception (Haxby et al., 2000). However, the IPS has been found to be more strongly activated when subjects selectively focus their attention on the gaze aspect of a stimulus face than when focusing on the face identity (Hoffman and Haxby, 2000). The FEF has been described to be involved in motor control and generating saccades (McDowell et al., 2008; Schall, 2009). It is supposed to receive input from various extrastriate areas and in turn exert top-down control on these areas (Schall, 2009). Nummenmaa et al. (2010) demonstrated that FEF and IPS show increased connectivity with the posterior STS when watching gaze shifts in contrast to opening/closing eyes. Moreover, it has been shown that direct gaze resulted in increased connectivity between FFG and amygdala, whereas averted gaze increased connectivity between FFG and IPS (George et al., 2001).

Overall, it is still in question if gaze and arrow cues are processed by different (Kingstone et al., 2004; Hietanen et al., 2006) or similar (Tipper et al., 2008; Sato et al., 2009) brain regions. Areas that have been associated with the processing of gaze cues are, among others, the STS, the FFG, the amygdala, the IPS, and the FEF. Watching a person shift his/her gaze might influence the functional connectivity between these areas (George et al., 2001; Nummenmaa et al., 2010).

Hence, the main aim of the present study was to further characterize the neural processing of social gaze and symbolic arrow cues, with emphasis on regions involved in the processing of meaningful social information (STS, FFG) and their connections to other brain regions (amygdala, IPS, FEF). In contrast to Sato et al. (2009) who described the commonalities underlying gaze and arrow cueing, this study particularly addressed the question whether there are differences in the activation patterns associated with gaze and arrow cues. Functional magnetic imaging (fMRI) was used to investigate a Posner-like cuing experiment (Posner, 1980) with averted gaze and laterally pointing line-arrow-configurations for direct comparison of both cue types (gaze > arrow, arrow > gaze). STS and FFG were *a priori* selected for region of interest (ROI) analyses. Hence, STS and FFG should be more strongly activated for gaze than for arrow cues. For a description of the networks underlying gaze and arrow cueing, a seed voxel connectivity analysis was conducted. Voxels from the STS and the FFG were set as seed regions. For the comparison of gaze and arrow cues, these regions were expected to show enhanced connectivity with other regions involved in gaze processing and spatial attention (FEF, IPS, and amygdala). To our knowledge, there is only one study that investigated connectivity during a gaze cueing task (Callejas et al., 2014). Whereas most studies used an intermediate stimulus-onset-asynchrony (SOA; for example, 300 ms), which has been shown to reliably elicit gaze cueing effects, the present study was conducted using two different SOA (100 and 800 ms) to counteract habituation effects. As it was expected that the two SOA impose differing demands on cue processing, behavioral and imaging results are reported separately for 100 and 800 ms SOA. Furthermore, in contrast to previous studies an event-related design and naturalistic gaze cues were used. It has been suggested that using line drawing faces might delay electrophysiological components in ventral and lateral regions and thus delay face processing (McCarthy et al., 1999).

## Materials and Methods

### Ethics statement

The study was approved by the Institutional Review Board of the University of Giessen and participants provided informed consent before participating in the study. The declaration of Helsinki was conformed.

### Participants

Thirty-one volunteers (15 females, mean age: 25 years, range: 20–32 years, SD: 3.6; 16 males, mean age: 25 years, range: 21–32 years, SD: 3.1) participated in the study. Twenty-nine participants were right-handed and all participants had normal or corrected-to-normal vision. Individuals with a history of neurological or psychiatric disease were excluded. Three participants had to be excluded because of technical problems. All participants gave their informed written consent to participate in the study.

### Apparatus

Functional magnetic imaging-data was collected using a Siemens Verio 3 Tesla MRT. T2*-weighted echo planar imaging was conducted [TR = 2800 ms, TE = 30 ms, 90° flip angle, 192 mm FOV, 64 × 64 matrix, 4.0 mm slice thickness, 30 slices (descending), 1 mm gap]. The experiment was controlled with Presentation computer program (Neurobehavioral Systems, Inc.,). The program was synchronized to the pulses of the MRI-Scanner so that the second pulse started stimulus presentation. Stimuli were presented on a 24′′ screen mounted near the tube opening of the MRT. The participants watched the screen through the reflection in an angled mirror on top of the head coil (viewing distance was 236 cm).

### Experimental stimulus displays

**Figure 1:**
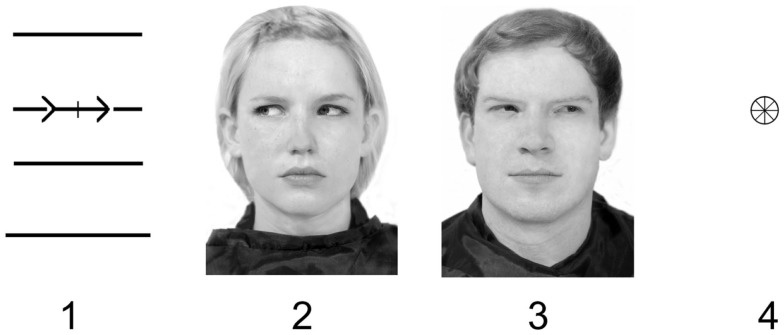
**Cue and target example stimuli [1 = arrow cue, 2 = gaze cue (female), 3 = gaze cue (male), 4 = target]**.

The gaze stimuli were gray-scale full-face photographs of one man and one woman, displaying neutral expressions. In these face displays gaze was averted for 30° to the right or to the left. The arrow cue depicted a geometric figure consisting of four horizontal lines and two arrows, both pointing either left or right. This stimulus was made to cover the same area as the gaze stimuli and, thus, keep the demands on visual analysis comparable. The visual angle subtended by the six cue stimuli was 2.8° horizontally and 4.8° vertically. The target stimulus depicted a small wheel-like circle. It appeared either right or left of the cue stimulus, subtending 0.3° horizontally. The distance between cue and target subtended 1° horizontally. The fixation cross and the target were black drawings. All stimuli were presented on a white background (Figure 1).

### Procedure

In the course of the experiment, each participant performed 400 trials (320 experimental trials and 80 catch trials). The trials were organized in four sections, each containing 100 trials. Sections were separated by three rest periods lasting for 30 s. One section was divided into 5 blocks of 20 trials each (16 experimental and 4 catch trials). The 16 experimental trials contained 2 trials of each combination of the experimental factors: cue type (gaze and arrow), SOA (100 and 800 ms), and congruency (congruent and incongruent). In these trials, the target appeared with the same frequency on the left and on the right side of the cue. Trials in which the target appeared on the cued side were termed congruent trials. When the target appeared on the uncued side the trial was termed to be incongruent. In catch trials no target appeared.

At the start of each trial, a fixation cross appeared on the screen for 1000 ms. Afterwards the cue stimulus appeared. Participants were presented with the gaze cue that matched their own gender. Following an interval of 100 or 800 ms (SOA), the cue was succeeded by the target. Both stimuli (cue and target) remained on the screen until the response of the participant but not longer than 1.5 s. In order to provide the same amount of time for the acquisition of fMRI-images for both SOA, each trial was extended by the time difference between the maximum trial length (3.3 s) and the time that passed between trial start and response. Catch trials were also extended to reach the maximum length of 3.3 s. Trials were followed by an inter-trial interval (ITI), which varied between 0 and 3000 ms. After each rest period, participants were presented with a short instruction, which lasted for 15 s.

Prior to the experiment participants were given a standardized instruction that introduced them to the task. Furthermore, they were provided with a practice version of the original task on a laptop computer. They received sufficient time to practice the task and the handling of the button device. It was emphasized that they should respond to the targets as fast and exact as possible by pressing a button on the button device with the index finger of their dominant hand. They were informed that the direction of the cue was not predictive of the target position. Prior to the beginning of the experiment a field map was recorded. Following the functional images T1-weighted anatomical images were acquired. The experiment lasted for about 34 min. The whole scanning session took about 1.5 h.

### fMRI-data

#### Whole-brain and region of interest analyses

Functional magnetic imaging-data was analyzed using SPM8 (Statistical Parametric Mapping, Wellcome Department of Imaging Neuroscience, London, 2009). The first three volumes were discarded to allow for magnetic saturation effects. The acquired images were corrected for differences in acquisition time (TA = 2.7067, reference slice = 15) and subject movement (twice the voxel size at maximum). Distortions caused by magnetic field inhomogeneity were corrected using the field map recorded prior to the experiment. To compensate for the individual variability in brain size and form the functional images were coregistered to each participant’s anatomical image (using normalized mutual information function) and fitted to the Montreal Neurological Institute (MNI; Lancaster et al., 2007) reference brain. Voxel size of the rewritten images was 3 mm × 3 mm × 3 mm. Finally, functional images were smoothed with a 9 mm FWHM Gaussian Kernel.

Statistical analysis was performed using the general linear model (GLM). Eleven regressors, modeling each combination of experimental variables (eight regressors), the catch trials (two regressors), and the rest period were included in the model. The six movement parameters obtained by the realignment procedure were entered as covariates. The onsets were time-locked to the cue onset. Regressors were convolved with the hemodynamic response function (HRF). Analysis generated statistical parametric maps, in which statistical parameters for each voxel were calculated. To identify brain regions that were activated more strongly by gaze or arrow cues, differential contrasts (gaze > arrow, arrow > gaze) were conducted. This was done separately for 100 and 800 ms SOA. A FWE-corrected alpha-level of 0.05 was chosen. Since at cue onset no information about target congruency was available to the participants and because this study focused on the differential effects of gaze and arrow cues, valid and invalid cues were not compared. To determine if the FFG and the STS were activated by gaze in contrast to arrow cues, ROI analyses for these regions using the small-volume-correction in SPM 8 (FWE-corrected, *p* < 0.05) were conducted. Again, this was done for 100 and 800 ms SOA separately. The mask for the FFG was taken from the SPM toolbox AAL (automated anatomical labeling; Tzourio-Mazoyer et al., 2002), implemented in the Wake Forest University (WFU) Pickatlas that provides a method for generating ROI masks based on the Talairach Daemon database (Lancaster et al., 1997, 2000; Maldjian et al., 2003, 2004). The STS mask was taken from Bischoff et al. (2007).

#### Analysis of “functional connectivity”

To analyze functional connectivity networks during gaze cueing, a seed region correlation approach was conducted [Alexander et al., 2012; Esslinger et al., 2009; He et al., 2007 in Supplementary Data; Klucken et al., 2012; Li et al., 2009; Meyer-Lindenberg, 2009; Toepper et al., 2014]. Connectivity is computed by extracting a reference time series from a chosen seed voxel and voxel-wise correlation of this time series with time series from all other voxels in the brain. The seed regions for this study (STS and FFG) were *a priori* selected, on the basis of previously published studies indicating their involvement in the processing of social information (see Introduction). The seed voxels were the 10 most highly activated voxels within a sphere of 10 mm radius around the peak voxel of each region identified by the aforementioned ROI analyses (100 ms SOA, gaze > arrow; 800 ms SOA, gaze > arrow). The seed voxel time series were high-pass filtered (128 s) and task-related variance was removed [Meyer-Lindenberg, 2009; He et al., 2007 in Supplementary Data]. Removal of task-related variance relies on reasoning that correlation due to task-related variance might only indicate simple coactivation of two brain structures, actually provoked by the task, however, independent of each other and without any connections between them. Correlation that survives removal of task-related variance indicates connectivity between the two structures because the observed residual covariation is assumed to be mediated by a brain network that might dynamically be built up by task demands. Accordingly, the first eigenvariate was calculated from the time courses. In order to remove spurious variance, eigenvariates from voxels within a white matter, a cerebrospinal fluid, and a brain mask were extracted. These noise eigenvariates were included into a whole-brain multiple regression SPM design as covariates of no interest, along with movement covariates for each person separately. The seed region eigenvariates were treated as covariates of interest. In a second level analysis, ROI analyses were performed on the connectivity data. Based on literature suggesting their involvement in gaze processing (see Introduction) the amygdala, the FEF, and the IPS were selected as ROI. The mask for the amygdala was taken from the AAL atlas (Tzourio-Mazoyer et al., 2002). Since the FEF has been found to be located in Brodmann Area 6 (McDowell et al., 2008; Schall, 2009), the corresponding mask implemented in the WFU Pickatlas was used. It has to be noted, however, that the mask of Brodmann area 6 extends beyond the FEF regions. The IPS mask was derived from Bischoff et al. (2007). Results reported in this study are based on positively correlated connections, not anticorrelated connections between seed regions and the aforementioned ROI. To analyze functional connectivity networks during gaze cueing, a seed region correlation approach was conducted (He et al., 2007; Esslinger et al., 2009; Li et al., 2009; Meyer-Lindenberg, 2009; Alexander et al., 2012; Klucken et al., 2012; Toepper et al., 2014). Connectivity is computed by extracting a reference time series from a chosen seed voxel and voxel-wise correlation of this time series with time series from all other voxels in the brain. The seed regions for this study (STS and FFG) were *a priori* selected, on the basis of previously published studies indicating their involvement in the processing of social information (see Introduction). The seed voxels were the 10 most highly activated voxels within a sphere of 10 mm radius around the peak voxel of each region identified by the aforementioned ROI analyses (100 ms SOA, gaze > arrow; 800 ms SOA, gaze > arrow). The seed voxel time series were high-pass filtered (128 s) and task-related variance was removed (He et al., 2007; Meyer-Lindenberg, 2009). Removal of task-related variance relies on reasoning that correlation due to task-related variance might only indicate simple coactivation of two brain structures, actually provoked by the task, however, independent of each other and without any connections between them. Correlation that survives removal of task-related variance indicates connectivity between the two structures because the observed residual covariation is assumed to be mediated by a brain network that might dynamically be built up by task demands. Accordingly, the first eigenvariate was calculated from the time courses. In order to remove spurious variance, eigenvariates from voxels within a white matter, a cerebrospinal fluid, and a brain mask were extracted. These noise eigenvariates were included into a whole-brain multiple regression SPM design as covariates of no interest, along with movement covariates for each person separately. The seed region eigenvariates were treated as covariates of interest. In a second level analysis, ROI analyses were performed on the connectivity data. Based on literature suggesting their involvement in gaze processing (see Introduction) the amygdala, the FEF, and the IPS were selected as ROI. The mask for the amygdala was taken from the AAL atlas (Tzourio-Mazoyer et al., 2002). Since the FEF has been found to be located in Brodmann Area 6 (McDowell et al., 2008; Schall, 2009), the corresponding mask implemented in the WFU Pickatlas was used. It has to be noted, however, that the mask of Brodmann area 6 extends beyond the FEF regions. The IPS mask was derived from Bischoff et al. (2007). Results reported in this study are based on positively correlated connections, not anticorrelated connections between seed regions and the aforementioned ROI.

## Results

### Behavioral data

On average, participants made 1% catch trial errors (button press in absence of target). Reaction time data from correct responses were collapsed across blocks and anticipations (RT < 100 ms), retardations (RT > 1500 ms), and omissions were excluded from the analysis. This accounted for 1.6% of the trials.

**Figure 2:**
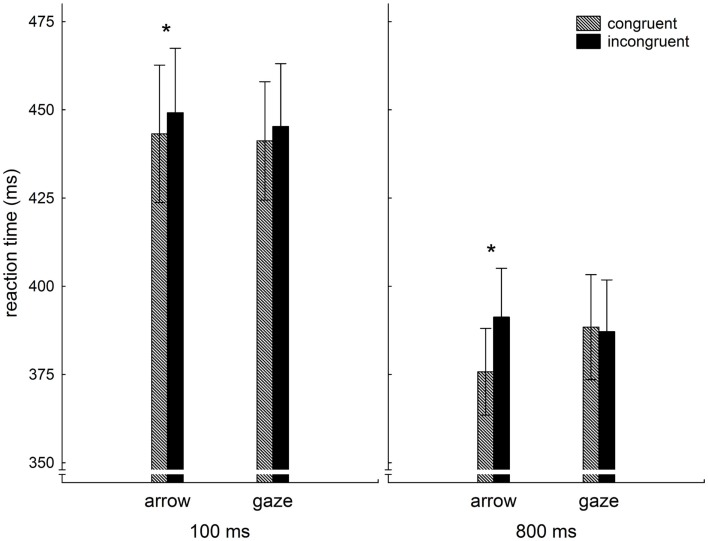
**Averaged median reaction times (ms) as a function of congruency and cue type in 100 and 800 ms SOA conditions**.

Behavioral data were analyzed using the statistical software package STATISTICA (Version 10, StatSoft). The median response latencies in each cueing condition were calculated for each participant. Median was used because it is more resistant to outliers. Next, the median response latencies for each cueing condition were averaged across participants. Results are presented in Figure 2.

To ascertain that both the laterally pointing arrows and the averted gaze shifted the participant’s attention, the averaged median reaction times were fed into a 2 (cue type: gaze, arrow) × 2 (congruency: congruent, incongruent) × 2 (SOA: 100, 800) repeated measures ANOVA. The results showed a significant SOA × cue type × congruency three-way-interaction (*F*_1,30_ = 5.0, *p* < 0.04). This demonstrates that the effect of congruency on cue type was different for 100 and 800 ms, as can be seen in Figure 2. However, the strongest effect was the main effect congruency (*F*_1,30_ = 30.3, *p* < 0.001), indicating that, overall, congruent cues resulted in faster reactions as compared to incongruent cues. In addition, the two-way interaction cue type × congruency was significant (*F*_1,30_ = 9.9, *p* < 0.005), showing that the reaction time benefit from congruent cues is more pronounced for non-social cues.

In order to describe possible differences between short and long SOA, we computed, separately for both SOA, two-way repeated measures ANOVA with factors congruency and cue type. These analyses revealed that for 100 ms SOA the congruency main effect (*F*_1,30_ = 6.3, *p* < 0.02) was the only significant effect, whereas for 800 ms SOA the main effect congruency (*F*_1,30_ = 21.8, *p* < 0.001) was complemented by the significant two-way interaction cue type × congruency (*F*_1,30_ = 20.3, *p* < 0.001). These findings are further supported by analysis of simple effects (LSD test), which revealed that a benefit from congruent cues can statistically be confirmed only for arrow cues. It could also be proved that reaction times for congruent arrow cues at 800 ms SOA were significantly faster than reactions under any other condition.

### Functional magnetic resonance imaging

Analysis of fMRI-data was conducted in three steps: (i) BOLD-responses to gaze and arrow cues (gaze > arrow, arrow > gaze) were contrasted, (ii) ROI analyses for the FFG and the STS were conducted, and (iii) the functional connectivity of these regions was analyzed using a seed region approach.

#### Whole-brain analyses

**Figure 3:**
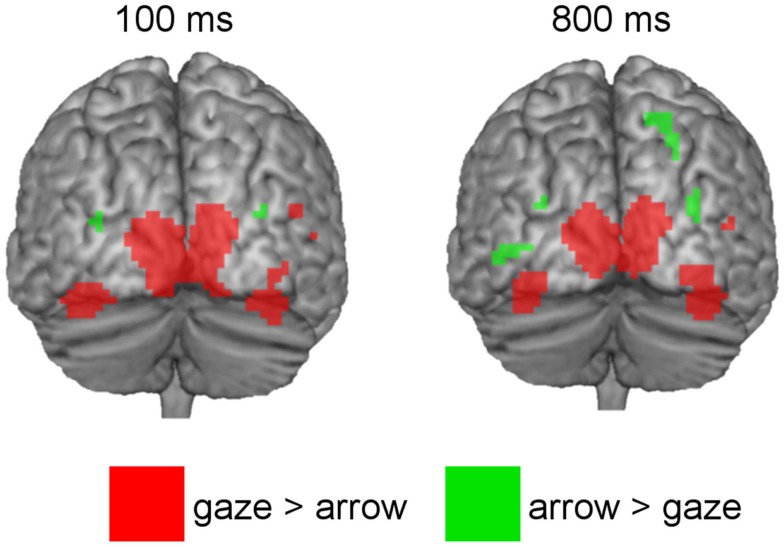
**Above threshold activations of brain regions showing greater BOLD-responses to directional gaze versus directional arrow cues (red) and to directional arrow versus directional gaze cues (green) at 100 and 800 ms SOA**.

Coordinates, *t*- and *p*-values of cortical regions responding more strongly to gaze in contrast to arrow cues and to arrow in contrast to gaze cues are listed in Tables 1 and 2. For a graphical representation of the results see Figure 3.

**Table 1:** **Brain regions showing greater BOLD-response to directional cueing by gaze cues than to directional cueing by arrow cues (gaze > arrow) at 100 ms and 800 ms SOA, *p* < 0.05, FWE-corrected**.

Anatomical region	100 ms					800 ms				
	Peak MNI-coordinates			*t*	*p*-value (FWE-corr.)	Peak MNI-coordinates			*t*	*p*-value (FWE-corr.)
	*x*	*y*	*z*			*x*	*y*	*z*		
Left SOG	−9	−97	4	9.99	0.000	−9	−100	7	11.06	0.000
Right CUN	12	−100	7	9.89	0.000	12	−97	10	12.68	0.000
Left FFG	−36	−85	−17	9.77	0.000	−39	−58	−17	9.89	0.000
	−39	−55	−20	8.84	0.000	−36	−82	−14	8.72	0.000
Right FFG	39	−46	−20	9.03	0.000	39	−49	−20	11.02	0.000
	–	–	–	–	–	33	−76	−11	7.61	0.000
Left CAL	−6	−94	−5	8.69	0.000	−3	−94	−5	8.16	0.000
Right CER	33	−70	−20	7.08	0.001	–	–	–	–	–
Right IOG	42	−79	−11	6.33	0.008	–	–	–	–	–
Right MTG	48	−61	16	4.72	0.027	51	−49	10	6.01	0.019
	57	−46	7	5.56	0.048	–	–	–	–	–

*Coordinates reflect positions relative to the MNI atlas (Montreal Neurological Institute, QC, Canada); FWE, family wise error; SOG, superior occipital gyrus; CUN, cuneus; FFG, fusiform gyrus; CAL, calcarine; CER, cerebellum; IOG, inferior occipital gyrus; MTG, middle temporal gyrus*.

**Table 2:** **Brain regions showing greater BOLD-response to directional cueing by arrow cues than to directional cueing by gaze cues (arrow > gaze) at 100 ms and 800 ms SOA, *p* < 0.05, FWE-corrected**.

Anatomical region	100 ms					800 ms				
	Peak MNI-coordinates			*t*	*p*-value (FWE-corr.)	Peak MNI-coordinates			*t*	*p*-value (FWE-corr.)
	*x*	*y*	*z*			*x*	*y*	*z*		
Left MOG	−33	−85	13	4.83	0.017	−45	−70	−2	7.54	0.001
	–	–	–	–	–	−30	−85	19	6.46	0.007
Right MOG	36	−82	19	4.78	0.021	36	−85	16	5.17	0.004
Right SPG	–	–	–	–	–	21	−70	55	6.81	0.003
Right SOG	–	–	–	–	–	27	−67	43	6.39	0.008

*Coordinates reflect positions relative to the MNI atlas (Montreal Neurological Institute, QC, Canada). FWE, family wise error; MOG, middle occipital gyrus; SPG, superior parietal gyrus; SOG, superior occipital gyrus*.

The gaze > arrow contrast showed significant activation at both SOA. For the 100 ms SOA areas of activation were located in temporal–occipital regions. Significant activation was observed in the superior occipital gyrus and the calcarine region of the left hemisphere. On the right hemisphere, the cuneus and the inferior occipital gyrus showed significant activation, as well as two areas within the posterior part of the middle temporal gyrus. In addition, the bilateral FFG was significantly activated, with two areas of activation located in the left FFG and one area in the right FFG. There was also a small activation in the cerebellum.

For the 800 ms SOA, the results demonstrate significant activation in the left superior occipital gyrus and calcarine region, as well as in the right cuneus and in one area of the middle temporal gyrus. The FFG was significantly activated as well, exhibiting two areas of activation in each hemisphere. There was no significant activation in inferior occipital regions or in the cerebellum.

The arrow > gaze contrast also showed significant activation at both SOA. For the 100 ms SOA, this activation was limited to areas within the bilateral posterior middle occipital gyrus. For the 800 ms SOA, additional activation in one area of the middle occipital gyrus, close to the inferior occipital gyrus was found, as well as in superior occipital and superior parietal regions of the right hemisphere.

#### Region of interest analyses

Region of interest analyses revealed significantly higher bilateral STS activation for gaze in contrast to arrow cues at both SOA (gaze > arrow; Table 3 top). The ROI analyses also revealed significantly higher bilateral FFG activation for gaze in contrast to arrow cues at both SOA (gaze > arrow; Table 3 bottom).

**Table 3:** **Activation differences between gaze and arrow cues (gaze > arrow; ROI analysis, voxel-level, *p* < 0.05, FWE-corrected for multiple comparisons)**.

ROI	Laterality	100 ms					800 ms				
		Peak MNI-coordinates			*t*	*p*-value (FWE-corr.)	Peak MNI-coordinates			*t*	*p*-value (FWE-corr.)
		*x*	*y*	*z*			*x*	*y*	*z*		
STS	R	57	−46	7	5.56	0.002	51	−49	10	6.01	0.001
	L	−51	−52	13	4.89	0.009	−51	−52	16	4.87	0.010
FFG	L	−36	−82	−17	9.48	0.000	−39	−58	−17	8.89	0.000
	L	−39	−55	−20	8.84	0.000	−36	−82	−14	8.72	0.000
	R	39	−46	−20	9.03	0.000	39	−49	−20	11.02	0.000
	R	27	−85	−14	4.46	0.026	33	−76	−11	7.61	0.000

*FWE, family wise error; STS, superior temporal sulcus; R, right; L, left; FFG, fusiform gyrus. Search volume: 1492 voxels (STS), 957 voxels (FFG)*.

#### Functional connectivity

Seed regions for the connectivity analyses were determined by drawing a sphere of 10 mm radius around the peak voxels identified by the ROI analyses mentioned above. For the FFG, the peak voxels were −36/−82/−17 (100 ms SOA) and 39/−49/−20 (800 ms SOA). For the STS, the peak voxels were 57/−46/7 (100 ms SOA) and 51/−49/10 (800 ms SOA; all coordinates in MNI-space).

Results revealed increased connectivity between FFG and amygdala, as well as between FFG and IPS, during gaze in contrast to arrow trials at both SOA (Table 4). The higher connectivity found between FFG and BA6 at 100 ms SOA is most likely not located in the FEF, but more superior, in the left supplementary motor area and the right superior frontal gyrus. There was no increase in connectivity between FFG and BA6 at 800 ms SOA.

**Table 4:** **Differences between gaze and arrow cues in functional connectivity between fusiform gyrus and ROI regions (gaze > arrow, *p* < 0.05, FWE-corrected for multiple comparisons)**.

ROI	Laterality	100 ms					800 ms				
		Peak MNI-coordinates			*t*	*p*-value (FWE-corr.)	Peak MNI-coordinates			*t*	*p*-value (FWE-corr.)
		*x*	*y*	*z*			*x*	*y*	*z*		
AMY	R	–	–	–	–	–	33	−1	−17	6.45	0.000
BA6/FEF	L	−3	2	73	6.59	0.000	–	–	–	–	–
	R	9	32	61	5.33	0.000	–	–	–	–	–
	L	−3	20	64	4.88	0.018	–	–	–	–	–
IPS	R	30	−52	31	4.41	0.019	27	−70	31	6.14	0.000

*FWE, family wise error; AMY, amygdala; FEF, frontal eye fields; BA6, Brodmann area 6; IPS, intraparietal sulcus; R, right; L, left. search volume: 130 voxels (AMY), 1465 voxels (BA6), 883 voxels (IPS)*.

The same analyses for the STS demonstrate an increased connectivity between this region and BA6, as well as between the STS and the IPS, at both SOA (Table 5). The activation in BA6 is located bilaterally in the precentral gyrus, as well as in the right middle frontal gyrus. These areas of activation most likely correspond to the human FEF (Schall, 2009). The STS did not show increased connectivity with the amygdala for gaze in contrast to arrow cues at either SOA.

**Table 5:** **Differences between gaze and arrow cues in functional connectivity between superior temporal sulcus and ROI regions (gaze > arrow, *p* < 0.05, FWE-corrected for multiple comparisons)**.

ROI	Laterality	100 ms					800 ms				
		Peak MNI-coordinates			*t*	*p*-value (FWE-corr.)	Peak MNI-coordinates			*t*	*p*-value (FWE-corr.)
		*x*	*y*	*z*			*x*	*y*	*z*		
BA6/FEF	R	45	2	40	8.65	0.000	45	2	40	10.16	0.000
	R	36	2	31	6.15	0.001	33	5	49	6.27	0.000
	L	−45	−1	28	5.58	0.003	36	2	31	6.16	0.001
	R	36	8	49	5.37	0.005	−48	−1	28	4.80	0.023
IPS	R	36	−46	34	4.54	0.014	21	−46	34	5.65	0.001
	R	–	–	–	–	–	36	−43	43	4.69	0.010

*FWE, family wise error; FEF, frontal eye fields; BA6, Brodmann area 6; IPS, intraparietal sulcus; R, right; L, left. search volume: 1465 voxels (BA6), 883 voxels (IPS)*.

## Discussion

The main finding of the present study is that differences in the neural processing of social gaze and symbolic arrow cues indeed exist. Contrary to studies indicating similar processing mechanisms for gaze and arrow cues (Tipper et al., 2008; Sato et al., 2009), it was found that both cue types involved distinct areas more strongly than the other cue type. Another important finding is that STS and FFG show a differential coupling to brain areas implied in voluntary control of attention during gaze cueing.

### Behavioral results

Behavioral results showed a significant difference in reaction times between congruent and incongruent arrow cues at both SOA. These results are in agreement with the finding that arrow cues induce reflexive shifts of attention to a cued location. There was no significant difference between congruent and incongruent gaze cues at either SOA. This is in line with studies showing that gaze cueing is not a universal effect. A recent study selected participants for their main fMRI-experiment on the basis of a preceding behavioral experiment (Callejas et al., 2014). Seven participants had to be excluded because they did not show a cueing effect for gaze cues, seven other participants because they did not show a cueing effect for arrow cues and one participant because he/she did not show a cueing effect for either cue type. Some studies showed that the gaze cueing magnitude can be influenced by gender (Bayliss et al., 2005), self-reported autistic traits (Bayliss and Tipper, 2005), or political temperament (Dodd et al., 2011). In previous studies, it has been demonstrated that gaze cueing effects can be obtained with 100 ms SOA (100 ms, Quadflieg et al., 2004; Akiyama et al., 2008; Tipper et al., 2008; 105 ms, Friesen and Kingstone, 1998; 150 ms, Greene et al., 2009; 195 ms, Ristic et al., 2002), as well as with 800 ms SOA (700 ms, Driver et al., 1999; Akiyama et al., 2008; 1005 ms, Ristic et al., 2002; 1900 ms, Callejas et al., 2014). One can only speculate on the reasons for the missing gaze cueing effect in the present study. Compared to the only fMRI study, which is close to the present study in regard to cueing conditions and which demonstrates a significant gaze cueing effect (Engell et al., 2010), the present study used two SOA that were notably shorter or longer, respectively. In addition, the Engell et al. sample had a high female ratio whereas in the current study the gender ratio was balanced. This might also have contributed to the lacking gaze cueing effect because it is well known that gaze cueing tends to be more pronounced in females.

### Differences in the neural processing of social gaze and symbolic arrow cues

Imaging results reveal that the relative contrast gaze > arrow increased activation in a variety of occipital and temporal areas, whereas the reverse contrast evoked activation in occipital and parietal regions. Areas that demonstrated increased activation for gaze in contrast to arrow cues were found in the left superior occipital gyrus and calcarine region, as well as on the right hemisphere in the cuneus, the inferior occipital gyrus, the cerebellum, and the middle temporal gyrus. The FFG showed bilateral activation. The reverse contrast (arrow > gaze) revealed stronger activation in the bilateral middle occipital gyrus, the right superior parietal gyrus, and the right superior occipital gyrus for arrow cues. Even though gaze and arrow cues have been controlled for visual size, i.e., the respective visual scans paths have been brought into line, it should be noted that there are differences in luminance and complexity between both cue types, which could have contributed to the observed differences in visual brain areas.

This result pattern of differential activation in ventral occipito-temporal and dorsal occipito-parietal regions by gaze and arrow cues was also found by Engell et al. (2010). While targeting the reorienting processes triggered by invalid gaze and arrow cues, the authors reported that gaze cues, in contrast to arrow cues, activated extrastriatal visual areas, occipito-temporal regions, as well as inferior and middle frontal regions. Arrow cues in contrast to gaze cues activated the left parietal lobe, the postcentral gyrus and the precentral sulcus. Therefore, one can conclude that, despite all commonalities [see, for example, Tipper et al. (2008) and Sato et al. (2009)], gaze, and arrow cues engage brain regions differently. Interestingly, the effects of social cueing on cortical activation emerged despite the lacking gaze cueing effect on the behavioral level. It can be speculated that gaze cues provide, at least for some subjects, not only spatial information but also other social information. This might contribute to activation of cortical social areas, however, possibly prevents processing of the spatial information as would have been indicated by a significant gaze cueing effect.

#### Fusiform gyrus and superior temporal sulcus

The strong bilateral FFG activation found in the main analysis and the subsequent ROI analysis most likely reflects the enhanced processing of the face cue. While the FFG has been linked to face processing (Haxby et al., 2002), it has also been reported to contribute to gaze perception (George et al., 2001; Pelphrey et al., 2003; Mosconi et al., 2005; Nummenmaa and Calder, 2009; Nummenmaa et al., 2010). In the present study, it was found that the FFG was more strongly recruited by directional gaze than by directional arrow cues. This is in agreement with studies reporting that the FFG is more responsive to faces than to objects (Kanwisher et al., 1997; Kanwisher, 2000; Tong et al., 2000) and fits the results from Hooker et al. (2003). Some other neuroimaging studies found fusiform activation for gaze cues when subjects were presented with naturalistic looking face images (Sato et al., 2009; Callejas et al., 2014). On the contrary, neither Kingstone et al. (2004) nor Tipper et al. (2008) found a significantly greater response in the FFG for gaze cues. However, both of these studies used schematic or ambiguous stimuli as social cues. These cues might have been too abstract to elicit fusiform response, especially when considering that both stimuli did not depict faces but eyes only.

Another important face processing area is the STS, which showed increased activation for the gaze > arrow contrast in the ROI analyses. This finding is in line with results from Hooker et al. (2003) and Kingstone et al. (2004). It has been proposed that the STS is not only involved in the processing of directional eye gaze but also more generally in the processing of biologically significant cues (Hooker et al., 2003; Materna et al., 2008). Moreover, the STS might be susceptible to intentions conveyed by eye gaze (Mosconi et al., 2005). Therefore, increased STS activation might reflect the greater social significance of the gaze cue in contrast to the non-social arrow cue. Some of the neuroimaging studies that investigated gaze and arrow cueing did not find greater activation in superior temporal regions for gaze cues (Hietanen et al., 2006; Sato et al., 2009). Thus, it is important to notice that design and data-analysis of these studies were quite different from the approach of the present study. By directly comparing directional gaze and arrow cues, the present study avoided the use of any type of direct or “neutral” cue. Remarkably, Engell et al. (2010) demonstrated that direct gaze cues might not be useful baseline cues. However, when contrasting non-directional gaze and arrow cues, Hietanen et al. (2006) did obtain STS activation.

#### Other foci of activation

In the present study, occipito-temporal regions were mainly recruited by gaze cues, whereas arrow cues recruited occipito-parietal areas. Notably, due to the relative contrasts (gaze > arrow, arrow > gaze) assumptions about overlapping activations cannot be made. Occipital or occipito-temporal activation in response to gaze cues has been found consistently across studies (Hietanen et al., 2006; Tipper et al., 2008; Greene et al., 2009; Sato et al., 2009; Engell et al., 2010; Callejas et al., 2014).

In contrast to gaze cues, arrow cues enhanced activation in superior occipital and parietal regions (bilateral middle occipital gyrus, right superior occipital gyrus, and superior parietal lobule). Hietanen et al. (2006) and Sato et al. (2009) also found several regions that responded more strongly to arrow cueing than to gaze cueing. Sato et al. (2009) showed that the left superior parietal lobule was specifically activated for directional versus non-directional arrows. Superior parietal regions have been associated with voluntary attentional control (Yantis et al., 2002; Yantis and Serences, 2003; Behrmann et al., 2004; Grosbras et al., 2005; Hahn et al., 2006; Corbetta et al., 2008). Therefore, the stronger activation in this area in the present study might be in line with the assumption that the processing of arrow cues is more dependent on top-down-control than the processing of gaze cues.

The present results demonstrate that no cue type activated frontal regions more than the other cue type. Moreover, gaze cues did not activate parietal regions more than arrow cues. An explanation might be that in the present study both gaze and arrow cues contained directional information. Thus, any regions involved in the processing of this information might not show activation when contrasting the cue types.

### Analysis of “functional connectivity”

Since the functions of individual brain areas may vary depending on coactivated areas (Hein and Knight, 2008), seed voxel connectivity analyses were employed to further explore the neural context in which gaze cues are processed. In the present study, STS and FFG showed differential coupling with the selected regions of interest. The STS showed stronger connectivity with right IPS and bilateral FEF for directional gaze cues in contrast to directional arrow cues. The FFG showed stronger connections with the right IPS as well, but not with the FEF. Instead, the FFG showed increased connectivity with the right amygdala. These results show that even though frontal or parietal activation was not found in the main analysis, the regions found by contrasting gaze and arrow cues (STS, FFG) are functionally connected to parietal and frontal regions.

To date, there are not many studies that investigated functional connectivity in the context of gaze and arrow cues. Nummenmaa et al. (2010) found enhanced connectivity between the STS and several regions of the ventral and dorsal attention networks, including FEF and IPS, for gaze shifts in contrast to opened and closed eyes. In addition, the authors reported that the FFG demonstrated a similar pattern of connectivity. Another study (Callejas et al., 2014) found stronger connectivity of face processing regions with regions of the dorsal and ventral frontoparietal attention networks for gaze in contrast to arrow cueing. Specifically, they found that the STS was connected to the right inferior frontal junction and the posterior STS/temporoparietal junction area. The fusiform face area, a face-selective region within the FFG, was connected to several visual and attentional regions.

Frontal eye field and IPS are parts of the dorsal attention network proposed by Corbetta and Shulman (2002). This system is supposed to influence stimulus processing in the sensory cortex by generating top-down-signals (Corbetta et al., 2008; Vossel et al., 2012). In addition, FEF and IPS have been found for programing and controlling eye movements (Awh et al., 2006; McDowell et al., 2008). Since FEF and IPS did not show increased activation in the main analysis and since only the STS seed exhibited enhanced connectivity with both regions, it seems unlikely that their activation in the connectivity analysis can be explained solely by their role in generating saccades (Nummenmaa et al., 2010).

It is interesting that the FFG showed increased connectivity with the right amygdala. George et al. (2001) proposed stronger connectivity between FFG and IPS for averted gaze, which could be replicated in the present study, and stronger connectivity between FFG and amygdala for direct gaze. The authors interpreted this finding as evidence for the special social meaning of eye contact. In the present study, no direct gaze condition was employed. Gaze direction of the stimuli-faces was averted for 30° throughout the experiment. Amygdala activation has been linked to gaze processing (Wicker et al., 1998; Kawashima et al., 1999; Nummenmaa and Calder, 2009) and gaze monitoring (Hooker et al., 2003), with amygdala impairment leading to impaired attentional orienting in response to gaze but not arrow cues (Akiyama et al., 2007). Additionally, it was found that the FFG and the amygdala show a strong bidirectional connection, even more so during face perception (Herrington et al., 2011). This is in line with the present results, which show a stronger connectivity between FFG and amygdala during the gaze condition.

In the present study, STS and FFG were connected with mostly right hemispheric regions [for a similar result see Callejas et al. (2014)], supporting the assumption that the right hemisphere is dominantly involved in the processing of reflexive attention orienting in response to gaze shifts (Okada et al., 2012). Noteworthy, functional connectivity measures the temporal correlation between spatially distant areas and thus does not provide causal information.

### Effect of SOA

Since it was expected that 100 and 800 ms SOA elicit different processes, the imaging results were examined separately for both SOA. Though we expected different activation, the following effects are only descriptive since we did not directly compare the SOA conditions.

Gaze cues at 100 ms SOA activated the inferior occipital gyrus and the cerebellum more than arrow cues. There was no differential activation in these areas at 800 ms SOA. For arrow versus gaze cues the right superior occipital gyrus and the right superior parietal gyrus showed increased activation at 800 ms SOA, but not at 100 ms SOA. The effect of SOA is also apparent when examining the results of the functional connectivity analyses, since the right amygdala showed increased connectivity with the FFG only at 800 ms SOA. It has been assumed that gaze cueing at 100 ms SOA is reflexive, whereas at longer SOA voluntary processes take over (Driver et al., 1999; Langton and Bruce, 1999; Friesen et al., 2004). This fits the result that the superior parietal lobule, which has been implicated in top-down-control, shows activation for arrow > gaze cues only at 800 ms SOA. On the contrary, gaze > arrow cues at 800 ms SOA increased activation in occipito-temporal regions similar to those that were active at 100 ms SOA. Hence, it seems possible that the length of SOA affected the neural processing of gaze and arrow cues differently. This finding, though only descriptive, stresses the importance of SOA for the comparability of studies.

## Conclusion

These findings support the view that the processing of social gaze and symbolic arrow cues is supported by at least partly different neural systems. By directly contrasting gaze and arrow cues, a clear differentiation in neural activation between gaze and arrow cues could be shown. Gaze cues activated occipito-temporal areas, including FFG and superior temporal sulucs, more than arrow cues, whereas arrow cues increased activation in occipito-parietal regions more than gaze cues. This might contribute to the notion that arrow cues are more dependent on voluntary processes than gaze cues. Moreover, face processing regions (FFG, STS) showed enhanced interaction with parietal and frontal regions involved in the top-down-modulation of visual areas during trials with social cues. Thus, these results add further evidence to the assumption of different processing mechanisms for gaze and arrow cues.

## Conflict of Interest Statement

The authors declare that the research was conducted in the absence of any commercial or financial relationships that could be construed as a potential conflict of interest.
